# Celecoxib enhances the therapeutic efficacy of epirubicin for Novikoff hepatoma in rats

**DOI:** 10.1002/cam4.1487

**Published:** 2018-04-23

**Authors:** Tian‐Huei Chu, Hoi‐Hung Chan, Tsung‐Hui Hu, E‐Ming Wang, Yi‐Ling Ma, Shih‐Chung Huang, Jian‐Ching Wu, Yi‐Chen Chang, Wen‐Tsan Weng, Zhi‐Hong Wen, Deng‐Chyang Wu, Yi‐Ming Arthur Chen, Ming‐Hong Tai

**Affiliations:** ^1^ Center for Neuroscience National Sun Yat‐Sen University Kaohsiung Taiwan; ^2^ Institute of Biomedical Sciences National Sun Yat‐Sen University Kaohsiung Taiwan; ^3^ Division of Gastroenterology and Hepatology Department of Internal Medicine Kaohsiung Veterans General Hospital Kaohsiung Taiwan; ^4^ School of Medicine National Yang‐Ming University Taipei Taiwan; ^5^ College of Pharmacy & Health Care Tajen University Pingtung County Taiwan; ^6^ Department of Biological Sciences National Sun Yat‐sen University Kaohsiung Taiwan; ^7^ Division of Hepato‐Gastroenterology Department of Internal Medicine Chang Gung Memorial Hospital Kaohsiung Medical Center Chang Gung University College of Medicine Kaohsiung Taiwan; ^8^ Division of Nephrology Kaohsiung Veterans General Hospital Kaohsiung Taiwan; ^9^ Department of Internal Medicine Kaohsiung Armed Forces General Hospital Kaohsiung Taiwan; ^10^ Doctoral Degree Program in Marine Biotechnology National Sun Yat‐Sen University and Academia Sinica Kaohsiung Taiwan; ^11^ Department of Medical Research Kaohsiung Chang Gung Memorial Hospital Kaohsiung Taiwan; ^12^ Core Laboratory for Phenomics and Diagonstics Department of Pediatrics Kaohsiung Chang Gung Memorial Hospital Kaohsiung Taiwan; ^13^ Department of Marine Biotechnology and Resources Asia‐Pacific Ocean Research Center National Sun Yat‐Sen University Kaohsiung Taiwan; ^14^ Center for Stem Cell Research Kaohsiung Medical University Kaohsiung Taiwan; ^15^ Division of Gastroenterology Department of Internal Medicine Kaohsiung Medical University Hospital Kaohsiung Taiwan; ^16^ Department of Medicine Faculty of Medicine College of Medicine Kaohsiung Medical University Kaohsiung Taiwan; ^17^ Center for Infectious Disease and Cancer Research Kaohsiung Medical University Kaohsiung Taiwan; ^18^ Department of Microbiology and Immunology Institute of Medical Research and Institute of Clinical Medicine College of Medicine Kaohsiung Medical University Kaohsiung Taiwan

**Keywords:** Antitumor immunity, cancer stem cells, celecoxib, epirubicin, hepatocellular carcinoma

## Abstract

Epirubicin is a chemotherapy agent for hepatocellular carcinoma (HCC). However, the outcome of HCC patients receiving epirubicin remains unsatisfactory. Moreover, our previous study indicated that celecoxib suppresses HCC progression and liver cancer stemness. This study evaluated the potential of celecoxib to serve as a complementary therapy during epirubicin treatment. Cell proliferation, apoptosis, invasiveness, and anchorage‐independent growth were analyzed in hepatoma cells. Therapeutic efficacy was validated in rat orthotopic Novikoff hepatoma. After animal sacrifice, the antitumor mechanism of celecoxib and epirubicin combined therapy was investigated by histological analysis. Celecoxib enhanced the cytotoxic activity of epirubicin in HCC cells by promoting apoptosis. Besides, celecoxib potentiated the antineoplastic function of epirubicin in inhibiting the invasiveness and anchorage‐independent growth of HCC cells. Ultrasound monitoring showed that combined therapy was more potent than either therapy alone in perturbing HCC progression. Consistently, the size and weight of dissected HCC tissues from rats receiving combined therapy were smallest among all groups. HCC treated with combined therapy exhibited the highest prevalence of apoptotic cells, which was accompanied by reduced proliferating and angiogenic activities in tumor tissues. Moreover, the expression levels of cancer stemness markers (CD44 and CD133) and drug transporter MDR‐1 were significantly diminished in rats receiving combined therapy. Besides, celecoxib treatment increased the infiltration of cytotoxic T lymphocytes (CTLs) and reduced the number of regulatory T cells (Tregs), tumor‐associated macrophages (TAMs), and the expression of immune checkpoint PD‐L1 in HCC tissues during epirubicin therapy. Celecoxib augmented the therapeutic efficacy while modulated cancer stemness and antitumor immunity. Thus, celecoxib may serve as complementary therapy to improve the outcome of patients with advanced HCC during epirubicin treatment.

## Introduction

Hepatocellular carcinoma (HCC) is the sixth most prevalent human malignancy worldwide 1. Current HCC therapies include surgery, liver transplantation, chemotherapy, transarterial chemoembolization (TAE), radiofrequency ablation, and sorafenib target therapy 2, 3. However, the overall prognosis for HCC remains poor.

Epirubicin is preferred over doxorubicin, one of the most popular anthracyclines, in some chemotherapy regimens as it appears to induce lower side effects 4. Epirubicin is like doxorubicin, and it can inhibit the activity of topoisomerase II alpha through inhibiting the cleavage of supercoiled DNA and suppressing DNA transcription and replication. Moreover, it has been used in the treatment of advanced HCC 5. Although the efficacies of many chemotherapeutic drugs have been validated in HCC treatment, the response rate is poor and ranges between 10% and 15%. Besides, no survival advantage has been displayed 6. Thus, the development of novel chemosensitizer is important for HCC control.

Celecoxib, a selective cyclooxygenase‐2 (COX‐2) inhibitor and nonsteroidal anti‐inflammatory drug (NSAID), is widely used for pain and inflammation. Celecoxib attenuates Akt phosphorylation and induces growth inhibition and apoptosis in HCC cells, which can be partially reversed by ectopic COX‐2 expression and prostaglandin E_2_ (PGE_2_) 7. Moreover, celecoxib downregulates drug pump MDR‐1 expression in human Hep3B liver cancer cells by a COX‐2‐dependent manner 8, and it also increases the intracellular accumulation of doxorubicin and enhances doxorubicin‐promoted cytotoxicity in MDA‐MB231 breast cancer cells 9. On the other hand, while chemotherapy effectively induces cancer cell apoptosis, associated PGE_2_ release by COX‐2 upregulation promotes neighboring cancer stem cells (CSCs) repopulation. This repopulation can be suppressed by celecoxib mediated blocking of PGE_2_ signaling. 10, 11. In the antitumor immunity, celecoxib treatment can promote the CD8^+^ CTL infiltration and reduce the FOXP3^+^ Treg recruitment in tumor tissues by IDO downregulation 12, 13. Moreover, celecoxib therapy can attenuate the M1 to M2 polarization and the PD‐L1 expression of TAMs 14, 15.

Our previous study showed that epirubicin treatment effectively delayed the tumor growth in orthotropic hepatoma model 16. Moreover, we have also found that celecoxib therapy potently suppressed HCC progression and extended the survival rate of HCC‐bearing rats through depletion of CD44/CD133 hepatic CSCs 17. But epirubicin or celecoxib monotherapy did not eradicate hepatic tumor and reduce tumor size of established hepatoma in our previous researches. It has been shown that the drug resistance by the enrichment of CSCs causes the poor response to chemotherapy in clinical HCC management 18. Thus, this study aims to evaluate the therapeutic potential and mechanism of celecoxib and epirubicin combined therapy for HCC control and provide the rationale for its beneficial use in HCC treatment.

## Materials and Methods

### Cell culture and drugs

Rat HCC N1‐S1, human HCC Huh‐7, and human HCC Hep3B cells were from Bioresource Collection and Research Center (BCRC); Hsinchu, Taiwan. Before shipping, these cells have performed STR‐PCR profile at BCRC. N1‐S1 cells were maintained in RPMI‐1640 medium (Gibco, Bethesda, MD) containing 10% calf serum (HyClone, Logan, UT, USA). Huh7 and Hep3B cells were maintained in DMEM medium (Gibco) containing 10% calf serum. All the media for cell culture were supplemented with 2 mM l‐glutamine (HyClone), 100 mg/mL streptomycin (HyClone), and 100 U/mL penicillin (HyClone). All cells were maintained under humidified conditions in 95% air and 5% CO_2_ at 37°C. Celecoxib was purchased from Pharmacia Corp (St. Louis, MO). Epirubicin was purchased from Sigma (St. Louis, MO). Sorafenib was purchased from Symansis (Temecula, CA).

### Cell proliferation assay

To access the growth rates, cells (5 × 10^3^/well) were seeded in 96‐well plates, and then cells were incubated overnight in 95% air and 5% CO_2_ at 37°C before drug treatment. After drug treatment for 48 h, alamarBlue reagent (10:1; Invitrogen, Carlsbad, CA, USA) was added and cells were incubated at 37°C for 2 h. Absorbance was measured with an ELISA reader (Dynex Technologies, Inc., Chantilly, VA) at 570–600 nm. Cell viability was expressed as a percentage of absorbance in treated wells relative to that of untreated (control) wells.

### Cell cycle analysis

Cells were treated with indicated concentration of celecoxib or epirubicin in complete medium for 72 h. Following treatment, cells were harvested and washed with PBS prior to fixation with ice‐cold ethanol (70%) and storage overnight at −20°C. Cells were washed twice with PBS prior to incubation with RNase A (10 mg/mL) and PI (50 mg/mL) for 30 min at 37°C. DNA content of 10,000 events was analyzed using a FACSCaliber flow cytometer (Becton Dickinson Biosciences, San Jose, CA) and the CELLQuest software (BD Biosciences, Franklin Lakes, NJ, USA).

### Colony formation assay

Huh‐7 and Hep3B cells (3000 cells per well) were treated with celecoxib for 10 days, and the colony (more than 50 cells) number was counted after fixing with paraformaldehyde and staining with crystal violet in 10% buffered formalin.

### Cell invasion assay

Huh‐7 cells were seeded in triplicate in the upper compartment of the chamber (2.5 × 10^4^ cells in 50 *μ*L per well) and supplemented with DMEM serum‐free media. The lower compartment was filled with 30 *μ*L of DMEM media containing 10% CS serum media. A polycarbonate filter (8‐*μ*m pore size Nucleopore; Costar, Cambridge, MA) was coated with 0.1% gelatin to allow cell adhesion, and the upper and lower compartments were separated by the coated filter. After incubation for 24 h in a humidified 5% CO_2_ atmosphere chamber at 37°C, cells on the upper side of the filter were moved to lower side. Migrated cells were fixed in absolute methanol and stained with 10% Giemsa solution (Merck, Germany). Finally, the fixed cells were photographed by microscope with digital images system (Olympus; Tokyo, Japan) and counted as mean ± S.D. per filter under five different high‐power fields.

### Animal experiments

All experimental procedures were reviewed and approved by the Institutional Animal Care and Use Committee at National Sun Yat‐Sen University (Kaohsiung, Taiwan). The ultrasound‐guided induction of Novikoff hepatoma in Sprague Dawley (SD) rats was performed as previously described 16. Briefly, rats (male, 6 weeks old) were implanted with N1‐S1 cells on day 0. After confirming HCC formation (with diameter larger than 5 mm) by US monitoring on day 10, rats (*n* = 32) were divided into four groups receiving: saline (by oral route; *n* = 8), celecoxib (30 mg/kg/day by oral route; *n* = 8), metronomic epirubicin (1 mg/kg/day by intravenous injection; *n* = 8), and combined epirubicin and celecoxib therapy. Based on the tumor diameters measured by ultrasound before and after various therapies, the disease status in animals was evaluated according to Response Evaluation Criteria in Solid Tumors (RECIST) ver.1.1 19. In brief, rats with an increase of 20% or more in tumor size or those with new tumors were regarded as having progressive disease (PD). Rats with a change in tumor size ranging from an increase of <20% to a decrease of <30% and with no new tumor were stratified as having stable disease (SD). Rats with a 30% or greater decrease in the target tumor were regarded as achieving partial response (PR). Rats with disappearance of the tumor were stratified as achieving complete response (CR).

### Immunohistochemistry and TUNEL staining

For immunohistochemical analysis, paraffin‐embedded hepatoma tissue blocks were sectioned into 3‐*μ*m‐thick slices and mounted on poly(lysine)‐coated slides. The slides were deparaffinized, blocked with 3% hydrogen peroxide for 10 min, and then subjected to antigen retrieval in 10 mM citrate buffer for 15 min in a microwave. The slides were incubated with CD31, CD44, CD133, and MDR‐1 antibody (Santa Cruz Biotechnology, Santa Cruz, CA, USA); Ki‐67 and PD‐L1 antibody (Thermo Fisher Scientific, Waltham, MA, USA); CD68 antibody (clone ED‐1) (EMD Millipore, Temecula, CA, USA); FOXP3 antibody (Bioss Antibodies, Woburn, MA, USA); and CD8 antibody (Biorbyt, Cambridge, UK), at 4°C overnight. After the sections were washed with PBS, they were incubated with horseradish peroxidase/Fab polymer conjugate (polymer detection system; Zymed Laboratories, South San Francisco, CA) for 30 min and detected using diaminobenzidine (1:20 dilution; Zymed Laboratories). TUNEL analysis was performed using the in situ Cell Death Detection Kit, Fluorescein (Roche, Indianapolis, IN), according to the manufacturer's protocol.

### Blood analysis

Peripheral blood was drawn from the tail veins of the rats after completion of treatment to check the white and red blood cells as well as the platelet counts by ADVIA 120 hematology system (Siemens Healthcare GmbH, Erlangen, Germany).

### Statistical analysis

Differences between the groups were statistically evaluated using the unpaired Student's *t* test. The results are presented as mean ± SD. All *P* value was two‐tailed, and *P *<* *0.05 was considered statistically significant. We used GraphPad Prism 7.0 (GraphPad Software, San Diego, CA) for the statistical calculations. The quantification of histological data was performed by ImageJ (NIH). The correlation of COX‐2 and CD44, CD133, CD68, or FOXP3 mRNA expression in TCGA HCC dataset was analyzed by UCSC Xena (http://xena.ucsc.edu/).

## Results

### Celecoxib augmented the antioncogenic efficacy of epirubicin in rat N1‐S1, human Huh‐7, and Hep3B hepatoma cells

**Figure 1:**
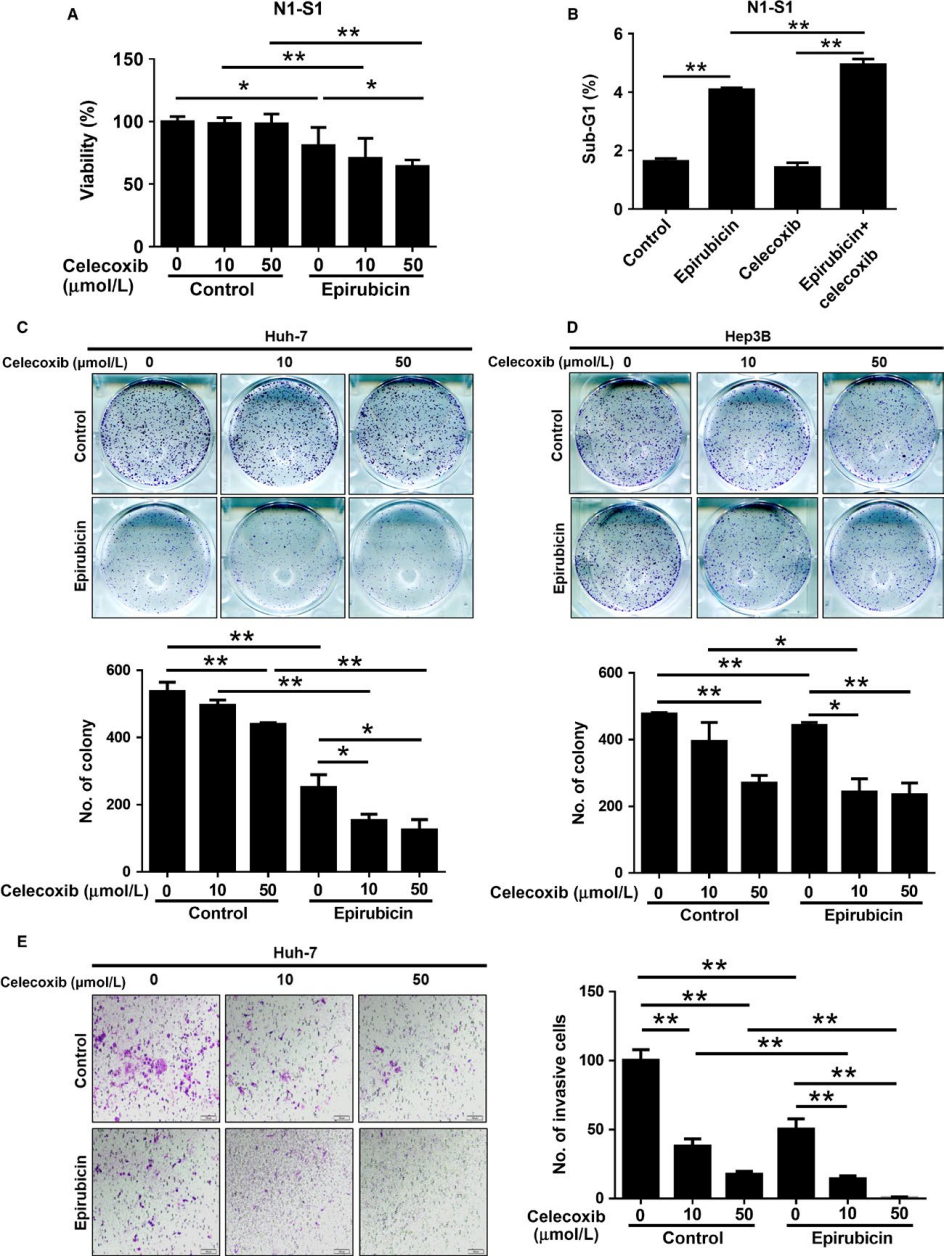
Celecoxib enhances the antitumor activity of epirubicin in vitro. (A) Cell proliferation analysis in N1‐S1 cells after celecoxib (10 and 50 *μ*mol/L), epirubicin (50 nmol/L), or combined treatment for 48 h. (B) The sub‐G1 fraction of N1‐S1 cells after celecoxib (10 *μ*mol/L), epirubicin (50 nmol/L), or combined treatment for 72 h was determined by flow cytometry. The anchorage‐independent growth of (C) Huh‐7 and (D) Hep3B cells after celecoxib (10 and 50 *μ*mol/L), epirubicin (50 nmol/L), or combined treatment for 10 days was determined by flat colony formation assay. (E) The cell invasiveness of Huh‐7 cells after celecoxib (10 and 50 *μ*mol/L), epirubicin (50 nmol/L) or combined treatment for 24 h was determined by invasion assay. Data were mean ± SD (*P <* *0.05, **P <* *0.01).

We first evaluate the complementary effect of celecoxib on the antineoplastic function of epirubicin in HCC cells. It was found that celecoxib significantly enhanced the antiproliferative effect of epirubicin in rat N1‐S1 hepatoma cells (Fig. 1A) and human Huh‐7 HCC cells (Fig. S1). Besides, flow cytometry analysis revealed that celecoxib treatment promoted the epirubicin‐induced apoptosis in rat N1‐S1 hepatoma cells (Fig. 1B). Moreover, application of celecoxib significantly augmented the epirubicin‐induced suppression of anchorage‐independent growth (Fig. 1C) and cell invasiveness (Fig. 1D) in human Huh‐7 and Hep3B HCC cells. Thus, these in vitro findings supported the potential of celecoxib in combination with epirubicin for HCC therapy.

### Serial ultrasound analysis revealed the potency of combined celecoxib and epirubicin therapy in suppressing Novikoff hepatoma in rats

**Figure 2:**
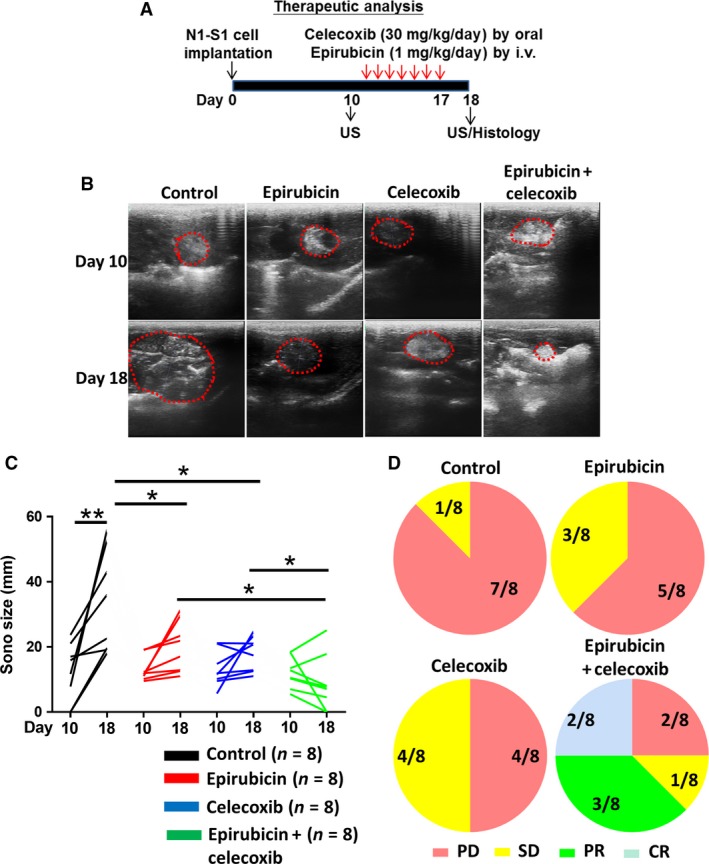
Oral celecoxib potentials therapeutic efficacy of epirubicin in rat orthotopic hepatoma model. (A) Experimental scheme. (B, C) US monitoring of rat Novikoff hepatoma before and after therapy (dotted line depicted the tumor areas). (D) RECIST analysis for the response of therapy. Data were mean ± SD (*P <* *0.05, **P <* *0.01).

Subsequently, we investigated the therapeutic efficacy of combination therapy using celecoxib and epirubicin in rats bearing established Novikoff hepatoma by serial ultrasound (US) analysis (Fig. 2A). When tumors were established on day 10, the animals were divided into four groups receiving the following: control, epirubicin, celecoxib, and combined celecoxib and epirubicin therapy. After a 7‐day treatment, ultrasound was performed to monitor HCC progression in animals before and after therapies. It was shown that either epirubicin or celecoxib therapy was effective in perturbing HCC progression (Fig. 2B,C). Noteworthily, combined celecoxib and epirubicin therapy was most potent in HCC suppression that the diameters of HCC receiving combination therapy were the smallest among all groups. This was supported using RECIST analysis, which revealed that either epirubicin or celecoxib therapy alone improved the diseases states and combined therapy group had the most promising outcome for HCC‐bearing rats (Fig. 2D). Thus, ultrasound studies suggested the potency of combined celecoxib and epirubicin therapy in rats with established HCC.

### Celecoxib enhanced the antiproliferative and proapoptotic function of epirubicin in Novikoff hepatoma

**Figure 3:**
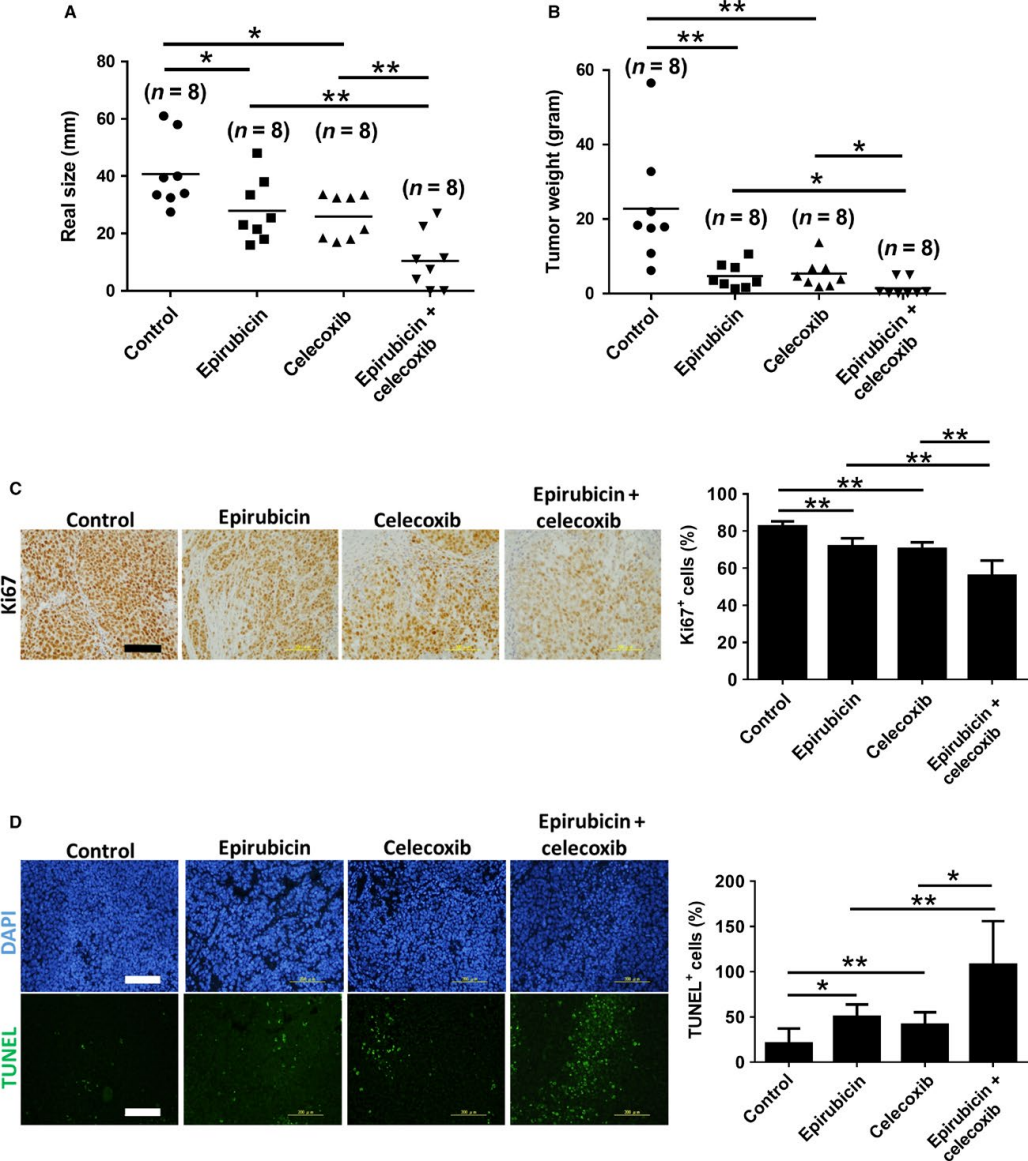
Celecoxib potentiates antiproliferative and proapoptotic ability of epirubicin in Novikoff hepatoma. (A) Caliper‐measured tumor size and (B) microbalance‐measured tumor weight after animal sacrificing. Histological analysis for (C) ki67 and (D) TUNEL from tumor tissues after therapy for 7 days. Data were mean ± SD (*P <* *0.05, **P <* *0.01). Scale bar = 200 *μ*m.

After ultrasound and RECIST analysis, the animals were sacrificed to dissect the HCC tissues for histological analysis. Consistent with ultrasound findings, the dissected HCC tissues from rats receiving combined celecoxib and epirubicin therapy were smallest in diameter (Fig. 3A) and weight (Fig. 3B) compared with other groups. These results validated that celecoxib enhanced the antineoplastic efficacy of epirubicin in HCC‐bearing rats by reducing tumor burden.

Immunohistochemical analysis of dissected HCC tissues was performed to elucidate the antineoplastic mechanism of combined celecoxib and epirubicin therapy for HCC. By Ki‐67 immunostaining, it was found that combined therapy significantly decreased the number of proliferating cells in HCC tissues compared with either therapy alone (Fig. 3C). Furthermore, TUNEL staining revealed that combined therapy significantly increased the number of apoptotic cells in HCC tissues compared with either therapy alone (Fig. 3D). These results indicate that celecoxib sensitizes HCC cells to epirubicin‐induced cytotoxic and apoptotic effects.

### Combined celecoxib and epirubicin therapy potently suppressed the neovascularization, hepatic CSC marker CD44/CD133, and MDR‐1 drug pump expression in Novikoff hepatoma tissues

**Figure 4:**
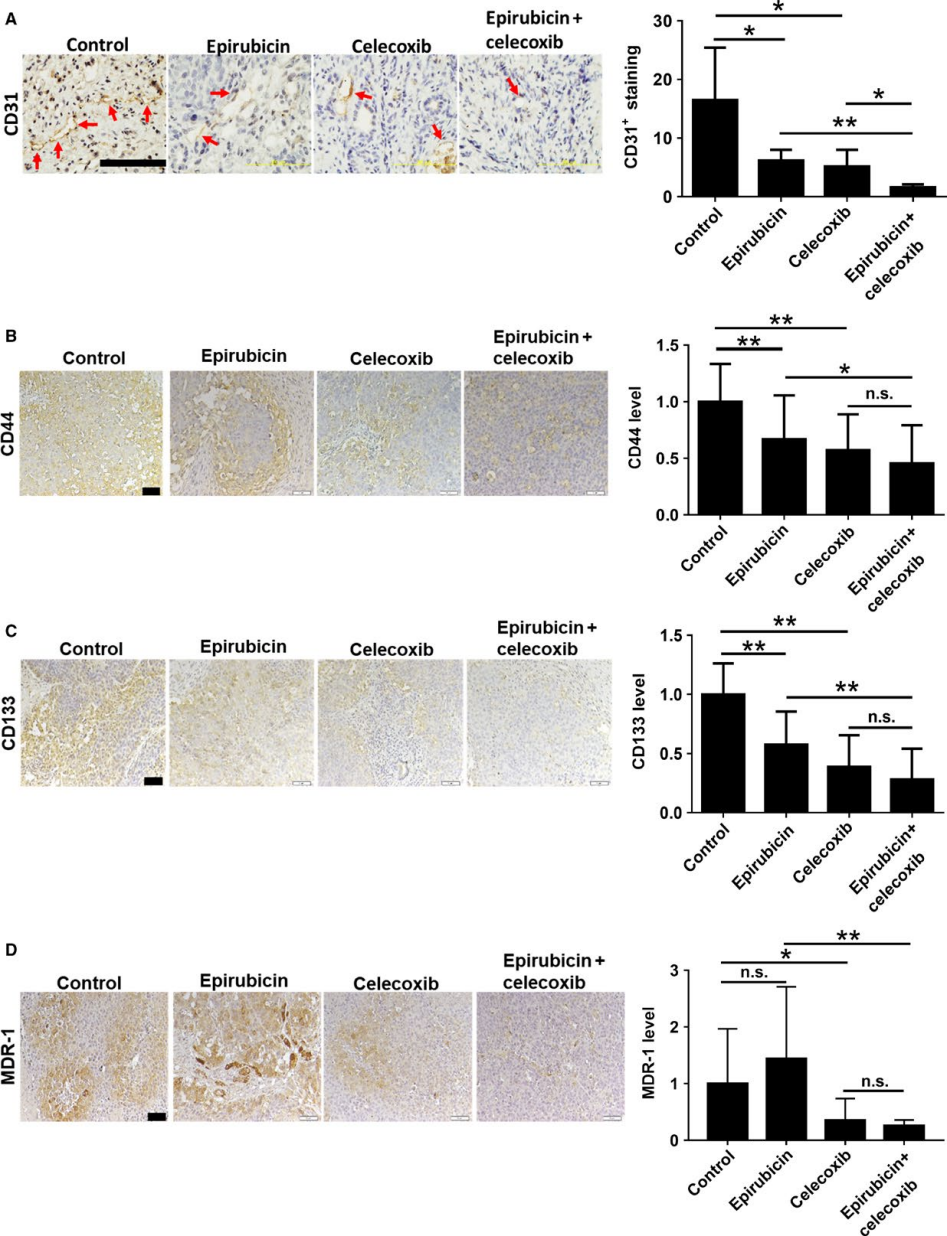
Epirubicin and celecoxib combined therapy blocks angiogenesis, cancer stemness, and drug efflux in Novikoff hepatoma. Immunohistochemistry analysis for (A) CD31, (B) CD44, (C) CD133, and (D) MDR‐1 from tumor tissues after therapy for 7 days (red arrow indicates blood vessels in the tumor tissues). Data were mean ± SD (n.s. = no significance, *P <* *0.05, **P <* *0.01). Scale bar = 200 *μ*m in CD31 analysis. Scale bar = 50 *μ*m in CD44, CD133, and MDR‐1 analysis.

Angiogenesis plays a pivotal role in tumor progression, metastasis, and recurrence. By analyzing neovascularization marker CD31, it was shown that combined therapy was more potent than celecoxib or epirubicin therapy in suppressing the tumor vasculature in HCC (Fig. 4A). Moreover, CD44 and CD133 are hepatic CSCs markers 20, 21, and CSCs can transdifferentiate into endothelial cells to promote tumor angiogenesis 22. We examined the expression profile of CD44 and CD133 in HCC tissues after treatment with various therapies. It was noted that epirubicin or celecoxib monotherapy reduced the protein levels of CD44 and CD133 in rat HCC tissues (Fig. 4B,C). Nevertheless, combined therapy remained most potent in depleting the CD44/CD133 expression in rat hepatoma. Furthermore, enhanced MDR‐1 expression in CSCs plays an important role in chemoresistance 23, and epirubicin is a high‐affinity substrate of MDR‐1 in many cancer cells 24. We found that celecoxib treatment significantly reduced the expression level of MDR‐1 in the rat hepatoma during epirubicin therapy (Fig. 4D). Together, combined therapy effectively inhibits the angiogenesis, cancer stemness, and drug efflux in HCC.

### Celecoxib did not worsen the epirubicin‐induced bone marrow suppression in HCC‐bearing rats

**Figure 5:**
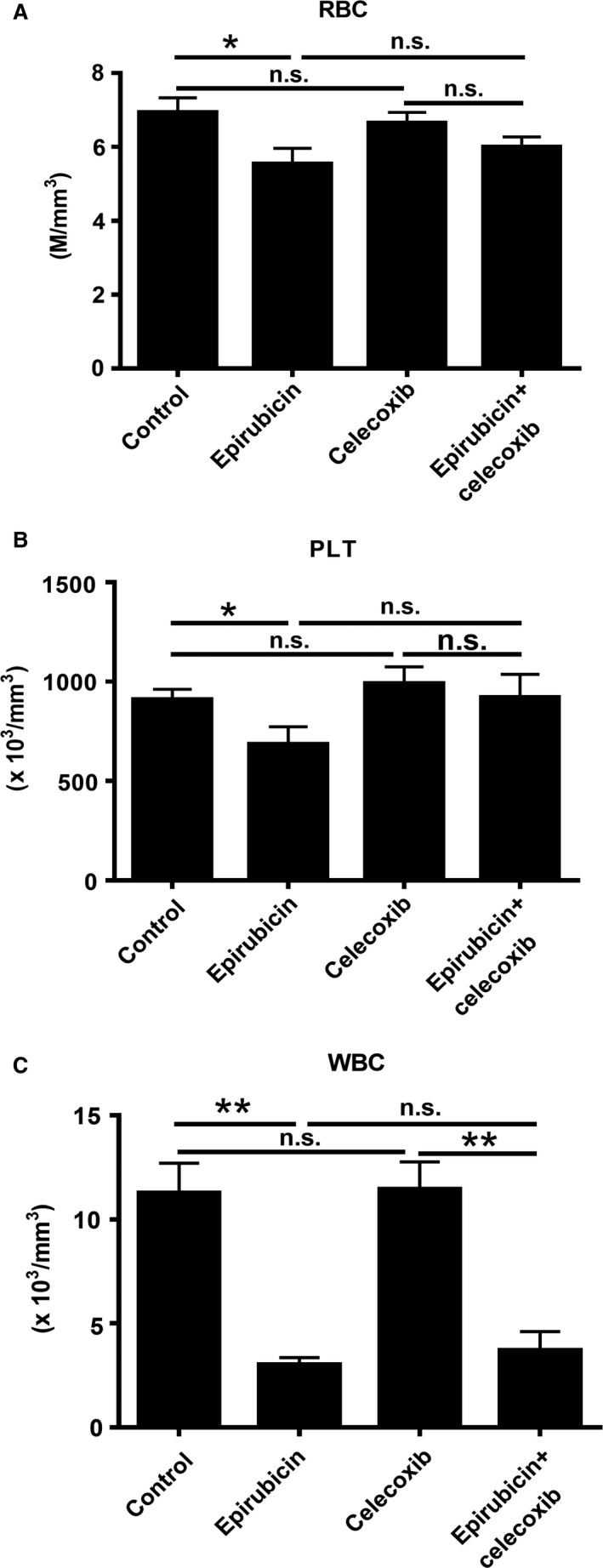
Celecoxib administration does not worsen the epirubicin‐induced bone marrow suppression in HCC‐bearing rats. (A) RBC, (B) PLT, and (C) WBC were analyzed after therapy for 7 days. Data were mean ± SD (n.s. = no significance, *P <* *0.05, **P <* *0.01).

One major adverse effect of chemotherapy is bone marrow suppression 25. Furthermore, it has been shown that celecoxib can suppress cell proliferation and arrest cell cycle in bone marrow‐derived stem cells via affecting expressions of cell cycle regulators 26. Thus, we investigated the blood cell profile in HCC‐bearing rats by complete blood count (CBC) analysis after various therapies. Indeed, the number of red blood cells (RBC), platelets (PLT), and white blood cells (WBC) in epirubicin‐treated rats was significantly decreased compared with control groups (Fig. 5), indicating the presence of bone marrow suppression in animals. Moreover, celecoxib did not significantly affect the basal CBC parameters and worsen the epirubicin‐induced bone marrow suppression in the HCC‐bearing rats. Thus, celecoxib did not show myelotoxicity in our study.

### Celecoxib activated antitumor immunity during epirubicin treatment in Novikoff hepatoma

**Figure 6:**
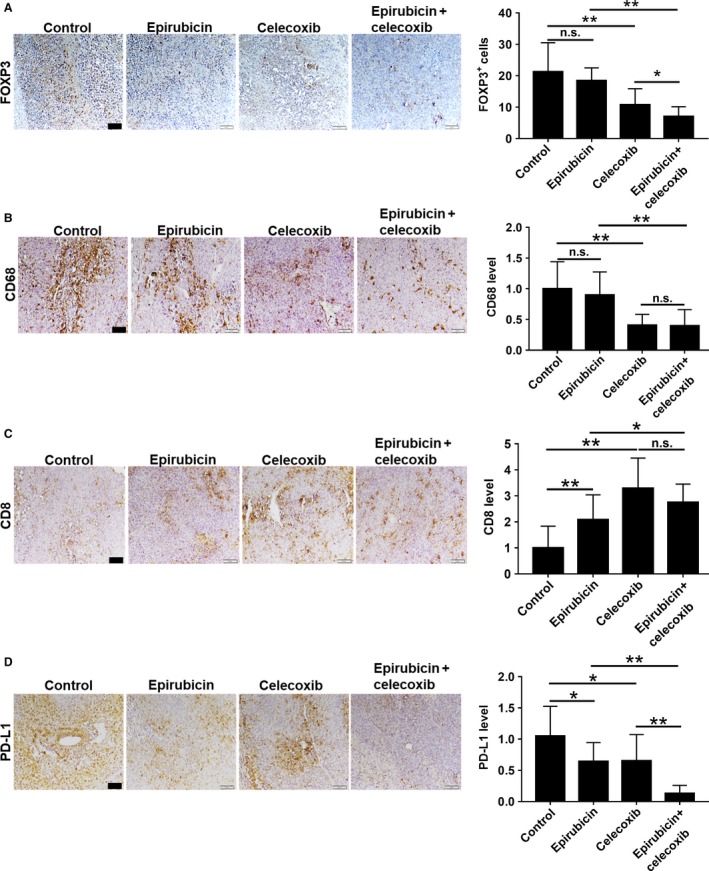
Celecoxib administration activates antitumor immunity in Novikoff HCC during epirubicin therapy. Immunohistochemistry analysis. (A) FOXP3, (B) CD68, (C) CD8, and (D) PD‐L1 from tumor tissues after therapy for 7 days. Data were mean ± SD (n.s. = no significance, *P <* *0.05, **P <* *0.01). Scale bar = 50 *μ*m.

Tregs and TAMs are immunosuppressive cells in tumor microenvironment, and they suppress antitumor CTL immunity in many cancers 27. Immune checkpoint PD‐L1 can modulate the immunosuppressive function of Tregs and TAMs 15, 28 and the CTL cytotoxicity in tumor tissues 29. Thus, we then investigated the immune‐modulatory effect of various therapies in Novikoff hepatoma by immunohistochemistry. It was found that epirubicin monotherapy increased PD‐L1 expression and CD8^+^ CTL infiltration, but it did not significantly affect Foxp3^+^ Treg and CD68^+^ TAM recruitment in Novikoff hepatoma (Fig. 6). Moreover, celecoxib administration significantly increased the infiltration of CD8^+^ CTLs, and it also reduced Foxp3^+^ Tregs, CD68^+^ TAMs, and PD‐L1 expression in rat HCC tissues during epirubicin therapy. Therefore, celecoxib complementary therapy activated antitumor immunity in tumor microenvironment, thereby halting HCC progression.

## Discussion

The present study has demonstrated for the first time that celecoxib potentiates the antitumor efficacy of epirubicin by suppressing the oncogenic behaviors of HCC cells in vitro. In rat Novikoff hepatoma model, celecoxib and epirubicin combined therapy was superior to either celecoxib or epirubicin alone in reducing tumor burden and improving diseases status. The advantage of combined therapy is further manifested by histological findings including antiproliferation, apoptosis induction, neovascularization blocking, CSCs inhibition, and antitumor immunity activation. Finally, celecoxib administration did not worsen epirubicin‐induced myelotoxicity. Given that celecoxib therapy is safe and well tolerated for HCC‐bearing animals 17, the present study supports the potential of combined epirubicin and celecoxib therapy for advanced or unresectable HCC.

In animal experiment, the most manifested growth‐inhibitory effect was achieved by combined therapy, and the size and weight of hepatoma with combined therapy were significantly smaller than them with epirubicin or celecoxib monotherapy. Although the efficacies of many chemotherapeutic agents have been tested in clinical HCC patients, the poor outcome and prognosis are still shown in the chemotherapy‐administrated patients 6. This may be due to the unbearable side effects of the conventional maximum tolerated dose (MTD) of chemotherapy upon patients who already have chronic liver diseases and cirrhosis 30. Moreover, some studies have reported epirubicin can induce bone marrow suppression and hepatotoxicity in animal studies 31, 32. Metronomic dose is not as toxic as the traditional maximum tolerated dose (MTD) of chemotherapy and better accepted by patients 33, 34. The metronomic dose of epirubicin in the present study is consistent with that in our previous study 16. In this study, metronomic epirubicin treatment still causes complete blood count (CBC) loss in peripheral blood. This indicated that low‐dose and high‐frequency chemotherapy regimen still caused serious myelotoxicity in HCC‐bearing rats. Furthermore, it has been reported that celecoxib can suppress cell proliferation and arrest cell cycle in bone marrow‐derived stem cells via affecting expressions of cell cycle regulators 26. Thus, whether celecoxib worsens the epirubicin‐induced bone marrow suppression is suspected. From the CBC parameters, no significant bone marrow suppression was showed in the celecoxib‐alone group, and celecoxib did not deteriorate the epirubicin‐induced myelotoxicity. This indicated that celecoxib administration showed high safety and tolerance in HCC‐bearing rats.

In histological analysis, celecoxib can enhance the antiproliferative, antiangiogenic, and proapoptotic function of epirubicin. Interestingly, single celecoxib treatment had no effect on the induction of cell apoptosis in vitro. This is not consistent with TUNEL staining result in tumor tissues. We mean that celecoxib interferes tumor microenvironment to regulate cell apoptosis, even cell proliferation, and angiogenesis. Some studies indicated that celecoxib can reduce the infiltration of regulatory T cells (Treg) 13 and alter the phenotype of tumor‐associated macrophages (TAMs) from M2 to M1 14. Tregs and TAMs can suppress antitumor CTL immunity in experimental mouse models 27. Celecoxib treatment also increases CD8^+^ CTL infiltration to tumor tissues via blocking of COX‐2‐IDO1 pathway 12. Moreover, recent study indicated celecoxib can inhibit immune checkpoint PD‐L1 in tumor microenvironment 15. From TCGA analysis, it was found COX‐2 expression was positively correlated with FOXP3 or CD68 expression in human HCC specimens (*n* = 423) (Fig. S2). In this study, we also found celecoxib administration can reduce FOXP3^+^ Tregs, CD68^+^ TAMs, and PD‐L1 expression and increase CD8^+^ CTLs in hepatoma tissues during epirubicin therapy. Interestingly, epirubicin monotherapy also decreased PD‐L1 expression, and increased CD8^+^ CTLs infiltration in hepatoma tissues. This indicated that metronomic chemotherapy modulated antitumor immunity in tumor microenvironment. The similar finding from recent report also demonstrated that metronomic chemotherapy can normalize tumor vasculature to increase tumor perfusion and oxygen pressure and improved perfusion alleviates hypoxia, which reprograms the immunosuppressive tumor microenvironment toward immunostimulation 35. Based on our finding, this immune‐competent orthotopic HCC model can reflect the truest experimental results and mimic the clinical condition. In future, we need more studies to clarify the effect of celecoxib and epirubicin on hepatic tumor microenvironment.

Serial chemotherapy can promote the enrichment of CSCs and tumor repopulation via COX‐2/PGE_2_ signaling in bladder cancer 10, and PGE_2_ can increase CD44^+^ and CD133^+^ CSCs subpopulation‐mediated EP4 signaling in colon cancer 36. From TCGA database, it was also found COX‐2 expression was positively correlated with CD44 or CD133 expression in human HCC specimens (*n* = 423) (Fig. S3). Our previous study also indicated celecoxib can suppress hepatoma progression and cancer stemness 17. In this study, hepatic tumor with combined therapy expressed least CD44 and CD133 CSCs markers. Interestingly, chemotherapy‐induced CSCs expansion was not shown in our study. On the contrary, chemotherapy reduced CD44 and CD133 expression in this rat orthotopic hepatoma. A similar finding from other research indicated metronomic chemotherapy increased the killing of CSCs via improved immune response 35. However, celecoxib can suppress hepatic cancer stemness and activate antitumor activity to enhance chemosensitivity in liver cancer.

One limitation of the present study is that the metronomic chemotherapy is not optimized, and significant bone marrow suppression showed in epirubicin‐treated rats. In the future, we need more studies to optimize the chemotherapy regimen. Moreover, epirubicin may cause heart failure 37, and the dose and frequency of epirubicin therapy should be considered carefully. On the other hand, celecoxib is known to suppress prostacyclin and predisposes patients to thrombosis 38, and maybe coadministration with low‐dose antithrombotic agent (eg, aspirin) can reduce celecoxib‐induced cardiac events. Besides, multiple kinase inhibitors sorafenib and regorafenib can prolong survival of advanced HCC patients 3, 39, but drug resistance has been shown in many sorafenib‐treated hepatoma cells and HCC patients 40. We also found that celecoxib can enhance sorafenib‐induced cytotoxicity in rat N1‐S1 and human Hep3B hepatoma cells (Fig. S4). Our finding showed that celecoxib improved the anticancer activity of chemotherapy, even target therapy in liver cancer. In the future, the combination of celecoxib and target therapy for HCC control will be expected, and more evidence is demanded to prove in subsequent study.

In summary, this report demonstrates that celecoxib improves the therapeutic efficacy of epirubicin plausibly via regulation of tumor vasculature, cell proliferation, cell apoptosis, cancer stemness, and antitumor immunity in immune‐competent orthotopic HCC model, and celecoxib and epirubicin combined therapy is well tolerable in rats. This study provides a rationale in use of celecoxib complementary therapy for HCC treatment.

## Conflict of Interest

The authors declare no conflict of interest.

## Supporting information


**Figure S1.** Celecoxib enhances the anti‐tumor activity of epirubicin in human hepatoma Hep3B cells.

**Figure S2.** COX‐2 expression is positively correlated with FOXP3 and CD68 expression in human HCC tissues.

**Figure S3.** COX‐2 expression is positively correlated with CD44 and CD133 expression in human hepatoma.

**Figure S4.** Celecoxib improves the anti‐tumor activity of sorafenib in vitro.
