# Supplementary figures and images for: Celecoxib enhances the therapeutic efficacy of epirubicin for Novikoff hepatoma in rats
Source: Cancer Med. 2018 Apr 23;7(6):2567–80. doi: 10.1002/cam4.1487 (PMC6010827; doi:10.1002/cam4.1487)

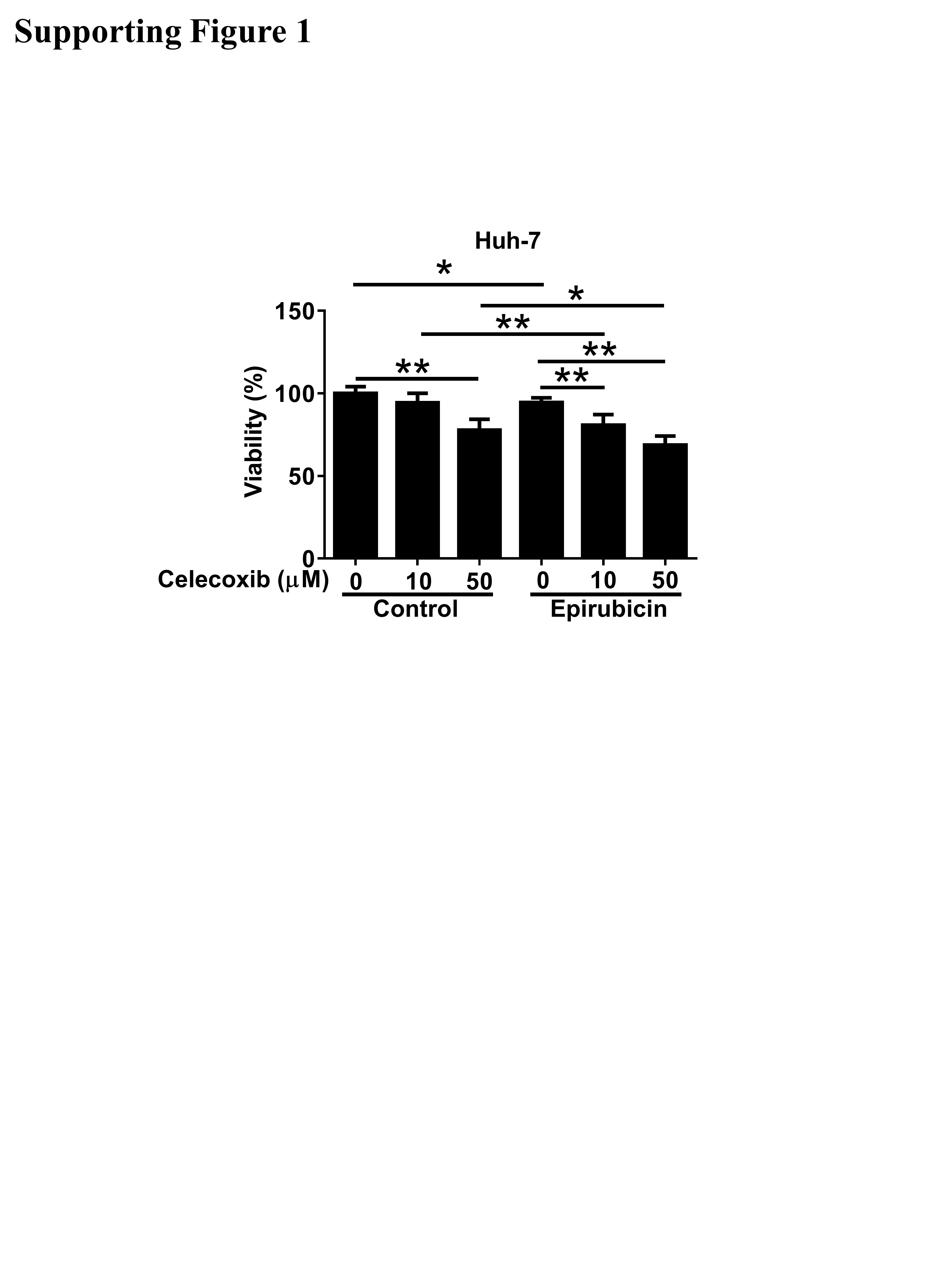

Supplement: Supplementary file 1 — Figure S1. Celecoxib enhances the anti‐tumor activity of epirubicin in human hepatoma Hep3B cells. [file CAM4-7-2567-s001.TIF]

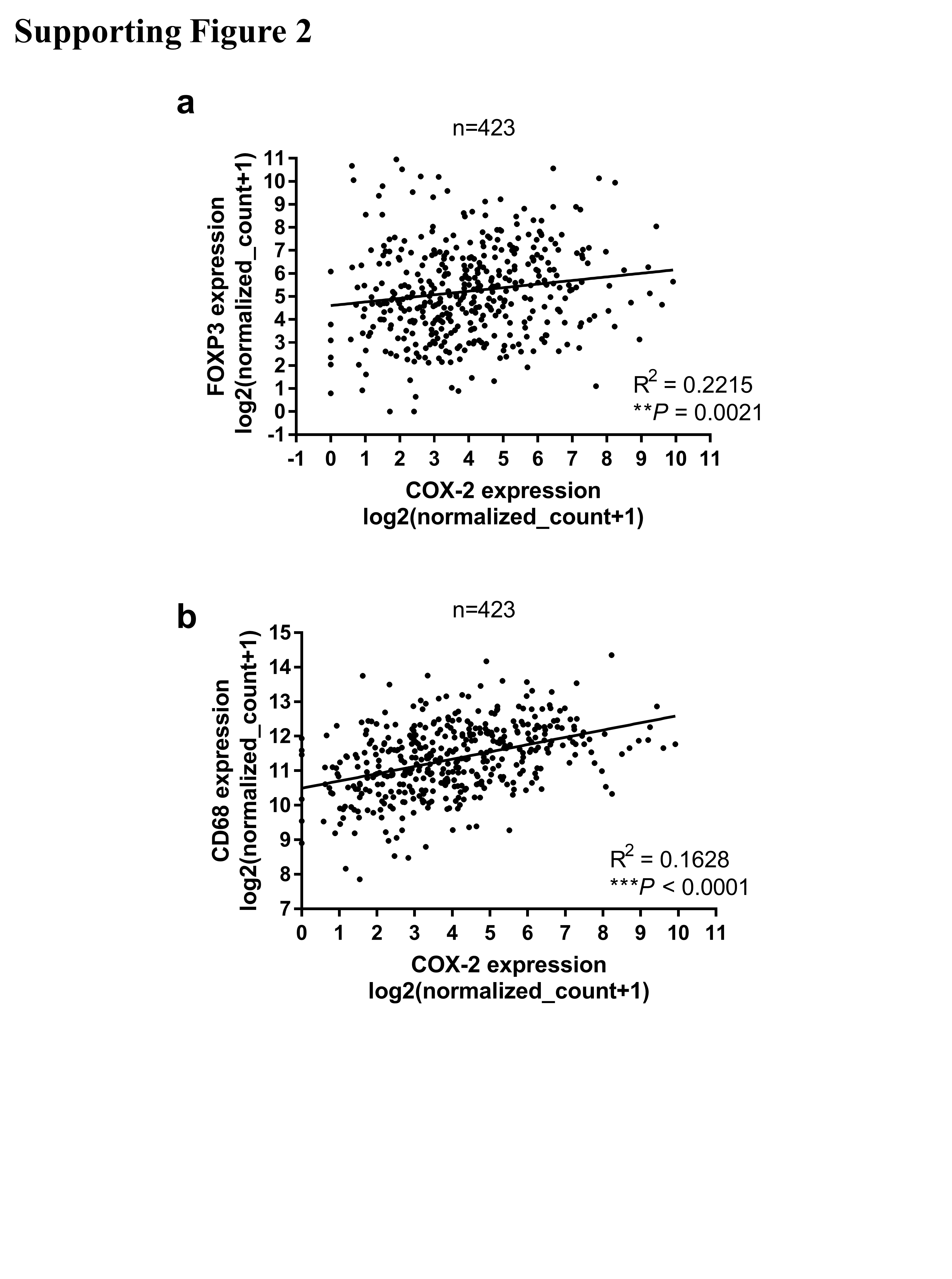

Supplement: Supplementary file 2 — Figure S2. COX‐2 expression is positively correlated with FOXP3 and CD68 expression in human HCC tissues. [file CAM4-7-2567-s002.TIF]

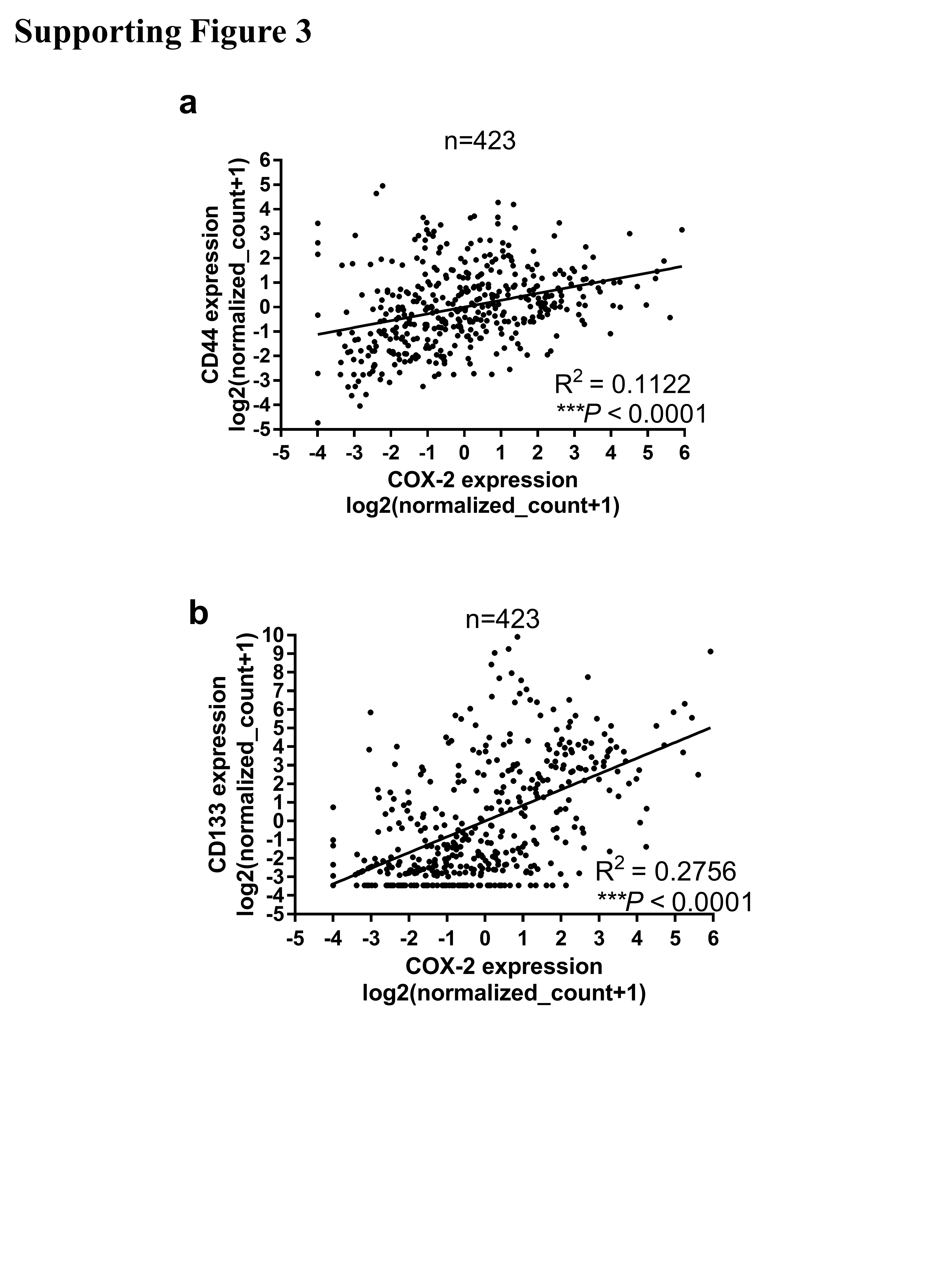

Supplement: Supplementary file 3 — Figure S3. COX‐2 expression is positively correlated with CD44 and CD133 expression in human hepatoma. [file CAM4-7-2567-s003.TIF]

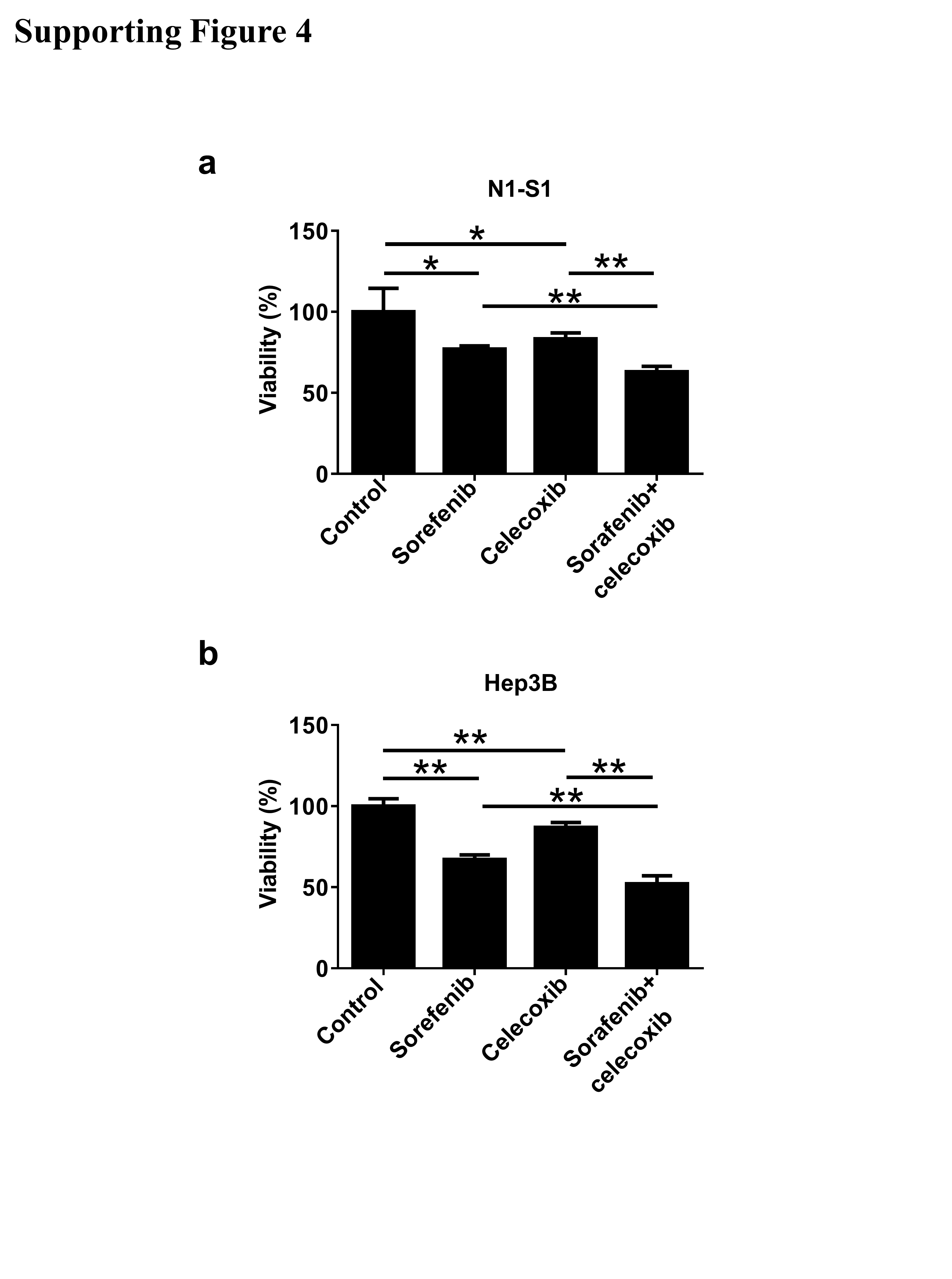

Supplement: Supplementary file 4 — Figure S4. Celecoxib improves the anti‐tumor activity of sorafenib in vitro. [file CAM4-7-2567-s004.TIF]
