# Supplementary material for: Celecoxib enhances the therapeutic efficacy of epirubicin for Novikoff hepatoma in rats
Source: Cancer Med. 2018 Apr 23;7(6):2567–80. doi: 10.1002/cam4.1487 (PMC6010827; doi:10.1002/cam4.1487)
Supplement: Supplementary file 5 [file CAM4-7-2567-s005.docx]

**SUPPLEMENTARY FIGURES AND LEGENDS**

**Figure S1.** Celecoxib enhances the anti-tumor activity of epirubicin in human hepatoma Hep3B cells. Cell proliferation analysis in Hep3B cells after celecoxib (10 and 50 μM), epirubicn (50 nM) or combined treatment for 48 h. Data were mean ± SD (**p* < 0.05, ***p* < 0.01).

**Figure S2.** COX-2 expression is positively correlated with FOXP3 and CD68 expression in human HCC tissues. TCGA analysis for the correlation of COX-2 expression and (a) FOXP3 or (b) CD68 expression in HCC patients.

**Figure S3.** COX-2 expression is positively correlated with CD44 and CD133 expression in human hepatoma. TCGA analysis for the correlation of COX-2 expression and (a) CD44 or (b) CD133 expression in HCC patients.

**Figure S4.** Celecoxib improves the anti-tumor activity of sorafenib *in vitro*. Cell proliferation analysis in (a) N1-S1 or (b) Hep3B cells after celecoxib (10 μM), sorafenib (10 μM) or combined treatment for 48 h. Data were mean ± SD (**p* < 0.05, ***p* < 0.01).
